# Spontaneous Minced Cartilage Procedure for Unexpectedly Large Femoral Condyle Surface Defect

**DOI:** 10.1155/2016/1498135

**Published:** 2016-07-18

**Authors:** G. M. Salzmann, G. A. Baumann, S. Preiss

**Affiliations:** Orthopaedics Lower Extremities, Musculoskeletal Centre, Schulthess Clinic, 8008 Zurich, Switzerland

## Abstract

Articular cartilage defects at the knee joint are being identified and treated with increasing frequency. Chondrocytes may have strongest potential to generate high-quality repair tissue within the defective region, in particular when large diameter defects are present. Autologous chondrocyte implantation is not available in every country. We present a case where we spontaneously covered an acute cartilage defect, which was significantly larger than expected and loose during initial arthroscopic inspection after reading preoperative MRI, by mincing the separated fragment and directly implanting the autologous cartilage chips into the defective region.

## 1. Introduction

Chondral and osteochondral lesions at the knee joint surface represent a severe medical as well as socioeconomic dilemma. If not treated well such lesions clearly predispose for early onset osteoarthritis (OA). Currently, many different surgical techniques to repair defective cartilage are on offer for the managing physician. The goal of cartilage repair surgery must be to generate tissue that is as close to the surrounding native tissue as possible as well as reversal of clinical symptoms. Current evidence is supportive of the fact that autologous tissue, in such, autologous chondrocytes, may result in the highest possible repair tissue within the defective region. Clinical outcome, return to sport, and long-term durability have been reported to be most satisfying following autologous chondrocyte implantation (ACI) when compared to other techniques [1]. At times the surgeon is intraoperatively subjected to different circumstances than expected following preoperative diagnostics. We present a case where the operative team was introduced into a large and acutely separated cartilage fragment.

## 2. Case Report

**Figure 1:**
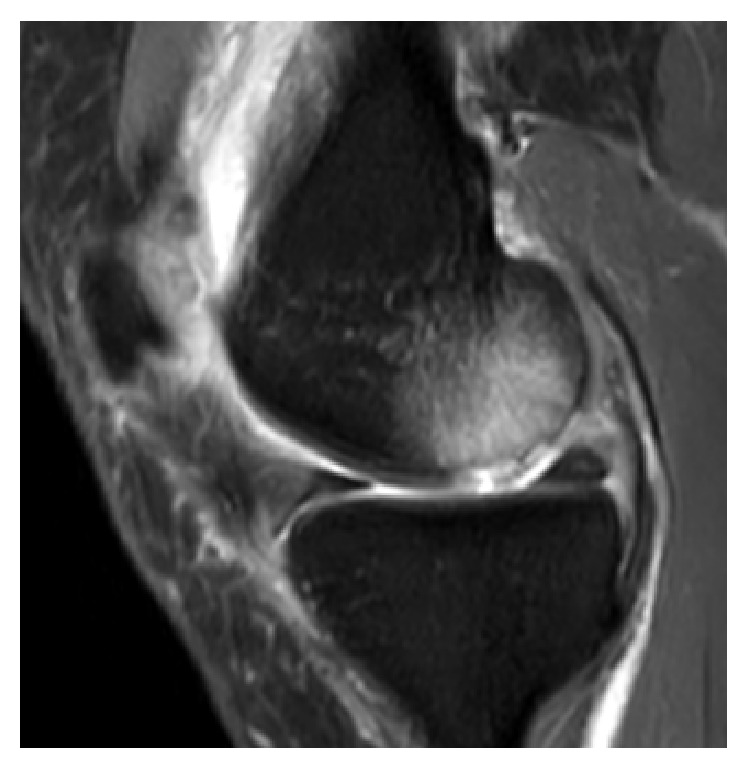
Sagittal T2-weighted MRI of left knee joint depicting cartilage lesion and large underlying BME at dorsomedial femoral condyle.

**Figure 2:**
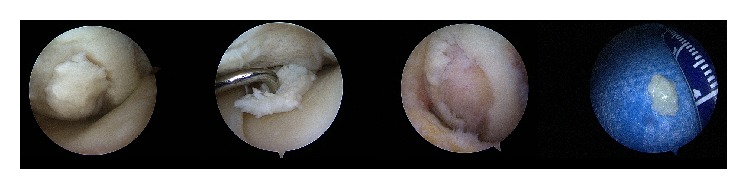
Intraoperative arthroscopic images in display of large separated fragment in situ, unstable under probing with remaining large cartilage lesion at dorsomedial femoral condyle after removal.

**Figure 3:**
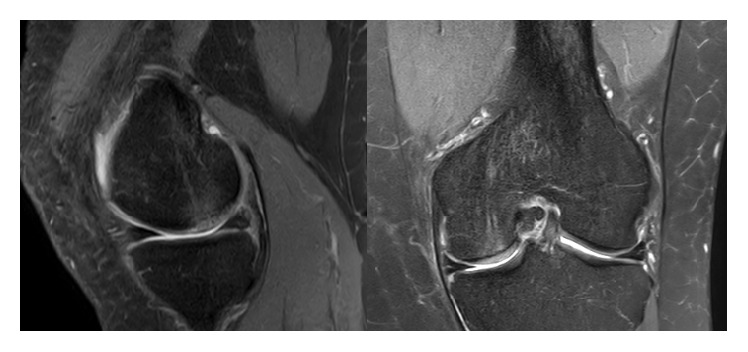
Sagittal and coronal T2-weighted MRI of left knee joint illustrating well repaired previous defective area with almost isointense, firmly integrated neocartilage formation at similar height to the surrounding cartilage without significant BME and healthy appearing subchondral bone at dorsomedial femoral condyle (6 months postoperatively).

A 53-year-old female presented to our outpatient clinic with rather acute chronic severe left-sided knee pain. The pain was not trauma related and had lasted for a few days. There were symptoms of locking. The knee joint has not been operated on before. Clinical findings confirmed a stable left knee joint with limited motion for full flexion as well as extension (110-5-0). Lachman, anterior, and posterior drawer were negative. The collateral ligaments were stable in full extension as well as in 30° of flexion. The patellofemoral alignment was normal and there was no apprehension sign. Clinical test for the menisci was negative (partly false positive for the anterior part of the medial meniscus). There was mild effusion and clear sharp pain at the medial femoral condyle. Conventional X-rays confirmed no significant pathology, no signs of advanced arthritis, and a straight mechanical axis (not shown). Subsequent magnetic resonance imaging (MRI) confirmed a large area of freshly appearing bone marrow edema (BME) at the dorsomedial femoral condyle with overlying highly irregular cartilage (Figure 1). The remaining joint appeared normal on MRI. After discussing the case with the patient we indicated to approach with knee joint arthroscopy first in order to inspect the medial condyle and debride the lesion plus potential antegrade drilling for relieve of the BME. During arthroscopy there appeared a large just recently separated pure chondral fragment at the dorsomedial femoral condyle with healthy appearing surrounding and opposing cartilage (Figure 2). The medial meniscus appeared intact. The remaining joint structures appeared intact. With regard to a recent separation and healthy appearing surroundings we decided to proceed with arthrotomy in order to repair the cartilage defect by mincing the healthy appearing cartilage piece. Following arthrotomy the large fragment could be retrieved easily. It was purely chondral. A refixation was deemed not promising. Consequently the large fragment with healthy appearing cartilage was minced into multiple small cartilage chips (<1 × 1 × 1 mm) using a scalpel at the back table. In parallel the defect was debrided to create a stable and healthy cartilage rim. The subchondral bone was intact. Defect dimensions after debridement were 2.5 × 1.5 cm and ICRS grade 3b. Yet, with regard to the BME seen on MRI, we frequently drilled into the subchondral bone at different locations and in different angles using a constantly water cooled 1.4 K-wire in antegrade fashion. Hereafter, the autologous chips were placed into the debrided lesion and fixed using fibrin glue. The chips had more than enough quantity to cover the lesion. After dehydration the joint was put through multiple full range of motion procedures. The repair tissue remained in place. Subsequently, the joint was closed in layers. Rehabilitation was performed as previously reported [2]. Following an uneventful postoperative course the patient presented without pain or locking sensations at our outpatient department at 6 weeks, 12 weeks, and 6 months postoperatively. Albeit no full muscular function, swimming and biking were already possible at last follow-up. Six-month MRI was in display of almost full regression of the BME and satisfying novel cartilage surface with good integration into the surrounding cartilage and subchondral bone. The transplant signal appeared almost isointense to the neighbouring cartilage (Figure 3). The calculated MOCART [3] score was 85 points. Lysholm score was 80 points. The patient was subjectively very satisfied with the procedure and would undergo it every time again.

## 3. Discussion

For treatment of joint surface pathology at the knee joint, one should aim for generating highest possible tissue within the previously defective region in order to return the patient to highest possible activity and protect the joint from premature osteoarthritis (OA). There are multiple technical options in order to treat symptomatic cartilage defects. Since 1994 it has been shown that autologous chondrocytes may generate highest tissue quality, also superior to concurring techniques with respect to clinical outcome [4, 5]. This is particularly true for large diameter defects over 3 cm^2^ as it is suggested by available guidelines [6]. Freshly separated purely chondral lesions, which are not amendable for refixation, constitute a good source for autologous chondrocytes [7]. With regard to intraoperative findings, purely chondral fragments are not recommended to be refixed [8]; we decided to proceed with an autologous minced cartilage procedure. Such technique has been proposed in the early 1980s already [9] and picked up again lately with promising early clinical data [10]. With regard to current increasing evidence, we felt confident to apply such technique [7, 11]. Furthermore, we were able to address underlying BME by simple antegrade drilling which in retrospect worked well. Yet, there is no current evidence to describe one surgical technique over the other when surgical BME management is regarded. It has to be considered that the antegrade drilling (drilled microfracture) might have in addition contributed to defect healing via an influx of stem cells. Incoming stem cells might have generated synergistic effects with surrounding cartilage chips, as it has been reported before [12]. The defect was considered too large for an isolated microfracture or osteochondral procedure. The newly forming cartilage over the subchondral bone generated good protection which was coupled by 8-week partial weight bearing in addition. We furthermore applied high doses of vitamin D over 3 months until lab values of vitamin D were highly normal. We consider here the described case as one example on how to potentially treat unexpected (and also planned), large, fresh, chondral lesions. High-quality, long-term studies with large patient cohorts would be required in order to provide better evidence.
